# Whole Blood mRNA Expression-Based Prognosis of Metastatic Renal Cell Carcinoma

**DOI:** 10.3390/ijms18112326

**Published:** 2017-11-03

**Authors:** Karthik V. Giridhar, Carlos P. Sosa, David W. Hillman, Cristobal Sanhueza, Candace L. Dalpiaz, Brian A. Costello, Fernando J. Quevedo, Henry C. Pitot, Roxana S. Dronca, Donna Ertz, John C. Cheville, Krishna Vanaja Donkena, Manish Kohli

**Affiliations:** 1 Department of Oncology, Mayo Clinic, 200 First Street SW, Rochester, MN 55905, USA; giridhar.karthik@mayo.edu (K.V.G.); ctsanhue@uc.cl (C.S.); Dalpiaz.Candace@mayo.edu (C.L.D.); Costello.Brian@mayo.edu (B.A.C.); Quevedo.Fernando@mayo.edu (F.J.Q.); Pitot.Henry@mayo.edu (H.C.P.); Dronca.Roxana@mayo.edu (R.S.D.); Ertz.Donna@mayo.edu (D.E.); 2 Biomarker Discovery, Center for Individualized Medicine, Mayo Clinic, Rochester, MN 55905, USA; Sosa.Carlos@mayo.edu (C.P.S.); KDonkena@mdanderson.org (K.V.D.); 3 Department of Health Sciences Research, Mayo Clinic, Rochester, MN 55905, USA; Hillman.David@mayo.edu; 4 Department of Oncology, Clínica Santa María, Santiago 8320000, Chile; 5 Department of Laboratory Medicine and Pathology, Mayo Clinic, Rochester, MN 55905, USA; Cheville.John@mayo.edu

**Keywords:** metastatic renal cell carcinoma, prognostic biomarkers, whole blood mRNA gene expression

## Abstract

The Memorial Sloan Kettering Cancer Center (MSKCC) prognostic score is based on clinical parameters. We analyzed whole blood mRNA expression in metastatic clear cell renal cell carcinoma (mCCRCC) patients and compared it to the MSKCC score for predicting overall survival. In a discovery set of 19 patients with mRCC, we performed whole transcriptome RNA sequencing and selected eighteen candidate genes for further evaluation based on associations with overall survival and statistical significance. In an independent validation of set of 47 patients with mCCRCC, transcript expression of the 18 candidate genes were quantified using a customized NanoString probeset. Cox regression multivariate analysis confirmed that two of the candidate genes were significantly associated with overall survival. Higher expression of *BAG1* [hazard ratio (HR) of 0.14, *p* < 0.0001, 95% confidence interval (CI) 0.04–0.36] and *NOP56* (HR 0.13, *p* < 0.0001, 95% CI 0.05–0.34) were associated with better prognosis. A prognostic model incorporating expression of *BAG1* and *NOP56* into the MSKCC score improved prognostication significantly over a model using the MSKCC prognostic score only (*p* < 0.0001). Prognostic value of using whole blood mRNA gene profiling in mCCRCC is feasible and should be prospectively confirmed in larger studies.

## 1. Introduction

Metastatic renal cell carcinoma is a heterogeneous disease encompassing multiple histologic subtypes and has variable outcomes. Since 2006, therapeutic advances have resulted in improvement of survival but identifying prognostic factors related to tumor biology in the metastatic stage is lacking [1]. Currently, prognostication is based on clinicopathologic prediction models, such as the Memorial Sloan Kettering Cancer Center (MSKCC) prognostic score [2], which does not incorporate tumor biology-based genomic information. Molecular expression profile signatures have enhanced prediction and prognostication in many tumor types and may also be able to improve current clinicopathologic measures of survival in metastatic renal cell cancer patients.

As renal cell cancer (RCC) is an immune-sensitive cancer, we hypothesized that alterations detected in the immune system of RCC patients could be used for prognostication of survival [3]. Furthermore, since global immune dysfunction is common [4,5,6], we hypothesized that changes detected in advanced RCC patients using next-generation sequencing of RNA extracted from whole blood can be used to determine prognosis [7]. A high throughput evaluation with broad coverage of gene expression of the whole blood extracted RNA, which primarily constitutes peripheral blood mononuclear cell fraction, was used to identify differentially expressed genes associated with survival. We used an initial cohort of metastatic RCC patients and an independent validation cohort to assess the prognostic ability of candidate gene expressions. We also compared it to the MSKCC prognostic score using overall survival as an endpoint.

## 2. Results

### 2.1. Patient Characteristics

Patient demographics, clinical, and treatment characteristics for both the discovery and validation sets are presented in Table 1. The discovery set (DS) included predominantly clear cell histology (16/19) and we restricted the validation set to only patients with clear cell RCC (47/47). In the DS, 7/19 samples were collected prior to start of systemic therapy and an additional 8/19 were collected soon after starting first-line systemic treatments for metastatic RCC. In the validation set (VS), all samples were collected prior to initiation of systemic treatment. The median duration of follow-up was 1.67 years in the DS and 2.72 years in VS, at which point 11/19 (58%) and 25/47 (53%) had died.

### 2.2. Sequencing Results of Discovery and Validation Sets

In the DS of 19 patients, the RNA integrity number (RIN) for each sample ranged from 8.6–9.8. The number of pair-end reads mapped (R1 and R2) for all the replicates corresponded to 34.6 to 61.0 million above 97% (Table S1). To select gene markers to discriminate between different conditions, we evaluated relative enrichment of genes in pairwise comparisons. Over 400 genes were statistically significant and we selected the top 18 genes for further validation (Table S2).

**Figure 1:**
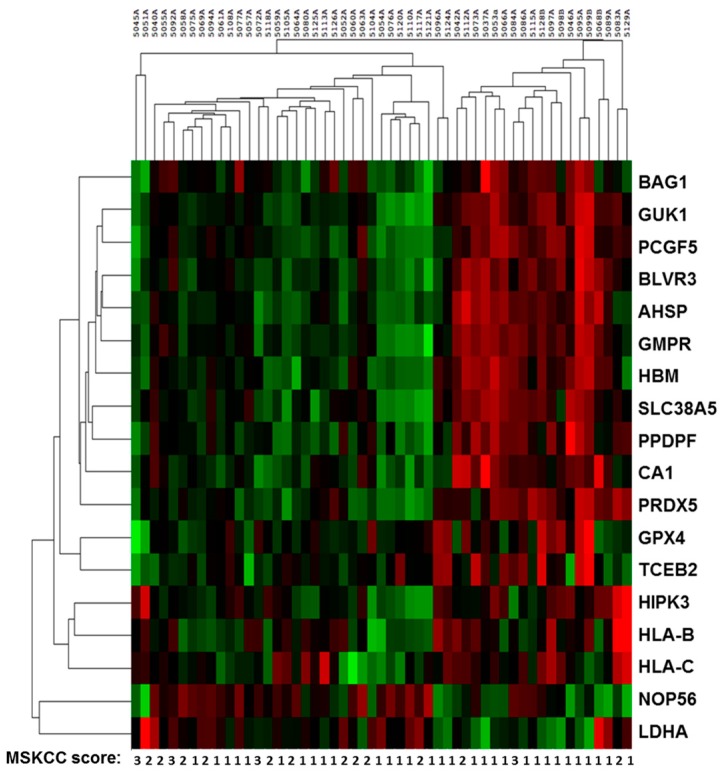
Heatmap of 18 targeted RNA transcript levels in the 47 patients from the validation set. Red indicates upregulation and green indicates downregulation. Labeled below is the MSKCC prognostic score for each patient.

To validate the association of mRNA expression with overall survival (OS), we performed gene expression profiling using NanoString technology in an independent cohort (validation set). We selected eighteen candidate genes for further prognostic performance based on the magnitude fold change of enrichment and statistical significance. We measured the expression of each gene using NanoString and normalized expression to four housekeeping genes (*ACTB*, *GAPDH*, *HPRT1*, *TBP*) [8,9,10]. All samples passed RNA extraction quality control criteria for optimal use with NanoString (ratio of 260/280 = 1.93–2.13 and RNA concentration = 79.2–396.2 ng/µL). Hierarchical clustering analysis showed that variations in gene expression inconsistently corresponded with MSKCC prognostic category (Figure 1).

In the validation set, we used Cutoff Finder [11] to select optimal threshold values and evaluated the hazard ratio, sensitivity, specificity, area under the curve (AUC), and statistical significance for each gene (Table 2). We confirmed that five of the eighteen genes retained strong prognostic significance. Higher expression of *BAG1* [hazard ratio (HR) of 0.22, *p* = 0.00044] and *NOP56* (HR 0.23, *p* = 0.0000210) were associated with better prognosis. Higher expression of *HLAB* (HR 2.32, *p* = 0.032), *GPX4* (HR = 2.57, *p* = 0.024), and *PRDX5* (3.55, *p* = 0.012) were associated with shorter survival. After Bonferroni correction, *HLAB*, *GPX4*, and *PRDX5* did not remain statistically significant.

### 2.3. Multivariate Analysis and Modeling for Prognosis in Metastatic Renal Cell Cancer

In multivariate analysis, two of the eighteen candidate genes remained significantly associated with OS: *BAG1* (HR 0.14, *p* < 0.0001, 95% CI 0.04–0.36) and *NOP56* (HR 0.13, *p* < 0.0001, 95% CI 0.05–0.34).

**Figure 2:**
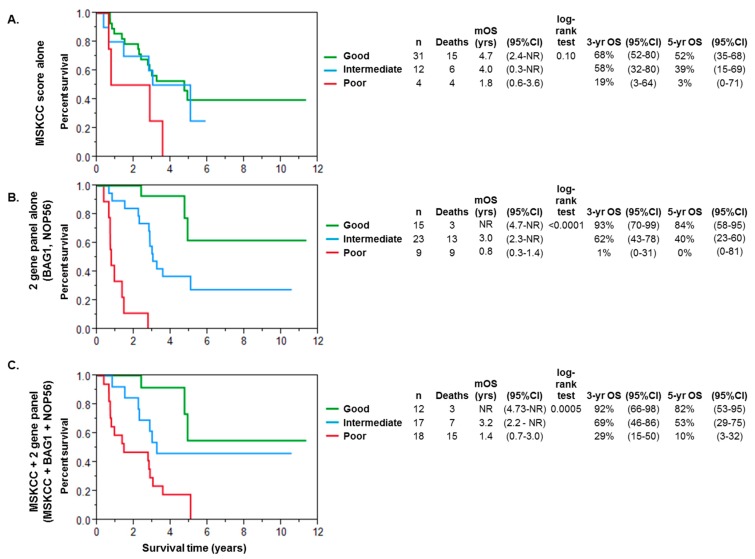
Kaplan–Meier survival analyses were generated from time of metastases to death or last follow-up in 3 scenarios: (**A**) using MSKCC score alone; (**B**) using the 2-gene panel alone at cutoffs specified in Table 2; or (**C**) the MSKCC score incorporating the 2-gene panel to identify good, intermediate, and poor risk categories. The number in each prognostic category, median overall survival (mOS) in years, 95% confidence intervals (CIs), log-rank test *p*-value, 3-year OS and 5-year OS for each scenario is are displayed in the adjacent table. NR = not reached. “n”-number of patients.

We also performed univariate analysis on clinical parameters and identified that metastatic disease at initial presentation was associated with survival. We found no associations with age, gender, Fuhrman grade, tumor stage, nodal stage, or sarcomatoid differentiation with clinical outcomes. When stratified by baseline MSKCC risk factors, the three-year OS was 68% in the good risk group (95% CI 52–80), 58% in the intermediate risk group (95% CI 32–80), and 19% in the poor risk group (3–64; Figure 2A). The multivariate Cox model incorporating BAG1 and NOP56 expression with the MSKCC score significantly improved prognostication of OS over the model using the MSKCC score alone (*p* < 0.0001).

We then evaluated our two-gene panel alone in the absence of clinical parameters to risk stratify patients into prognostic categories. Patients were categorized into good risk (elevated expression of both *BAG1* and *NOP56*), intermediate risk (elevation of either *BAG1* or *NOP56*), or poor risk (neither gene with elevated expression). When risk stratifying using our two-gene panel alone, three-year OS was 93% in the good risk group (95% CI 70–99), 62% in the intermediate risk group (95% CI 43–78), and 1% in the poor risk group (95% CI 0–31; Figure 2B).

Finally, we tested the predictive performance of a model incorporating expression of *BAG1* and *NOP56* into the MSKCC prognostic score. The baseline MSKCC was adjusted by subtracting one point for higher expression of *BAG1* or *NOP56*. Including our two-gene panel into the MSKCC score led to the identification of more poor risk patients, with fourteen patients being reclassified into a poor risk category, including five patients reclassified from good risk to poor risk. No patients were reclassified into a better prognostic category. Using the revised MSKCC score with incorporation of our two-gene panel, the three-year OS was 92% in the good risk group (95% CI 66–98), 69% in the intermediate risk group (95% CI 46–86), and 20% in the poor risk group (95% CI 15–50). While the MSKCC score alone was not significantly associated with OS (*p* = 0.10), the 2 gene panel (*p* < 0.0001) and the MSKCC + 2 gene panel (*p* = 0.0005) were prognostic for OS.

## 3. Discussion

We evaluated peripheral blood gene expression to identify novel prognostic biomarkers in patients with advanced clear cell RCC. Evaluating peripheral blood has many advantages. Obtaining these samples is safe and repeatable. Peripheral blood mononuclear cell-based immune response signatures may represent an easily accessible circulating tissue type for determining the host response to malignancy and treatments. When evaluating gene expression profiles in whole blood, an important consideration is that the extracted mRNA arises overwhelmingly from peripheral blood mononuclear cells. Circulating tumor cells (CTCs) may certainly be present, however even using appositive-selection-based method with RCC specific markers, the average number of CTCs captured were 6.5 per mL [12] compared to over 100,000 mononuclear hematopoietic cells per mL. While impossible to definitively state, we postulate that the gene expression profiles we captured arose from mononuclear blood cell gene expression opposed to circulating tumor cells.

Based on our observations made in the initial discovery set, we selected the top performing 18 candidate genes for further evaluation in an independent validation cohort comprised of pretreatment samples collected from 47 patients with clear cell RCC. In multivariate analysis, we confirmed that two genes (*BAG1* and *NOP56*) were of prognostic significance. When compared to the MSKCC prognostic score, which includes only conventional clinicopathologic parameters, our two-gene expression signature was better able to discriminate between prognostic groups. Increased expression of *BAG1* and *NOP56* had a protective effect and associated with a longer median overall survival. *BAG1* (BCL-2 associated anti-death gene 1) is a multifunctional protein that enhances the anti-apoptotic effects of Bcl-2 and coordinates signals to promote cell growth signals in response to cellular stress [13,14]. In hematopoietic stem cells, interleukin-2 induces upregulation of *BAG1* mRNA [15] and overexpression of BAG1 abrogated dexamethasone-induced apoptosis [16]. By promoting survival of hematopoietic cells, further investigation of *BAG1* in immune function and activity is warranted. NOP56 forms part of the box C/D small nucleolar RNAs (snoRNAs) that regulates posttranscriptional modification of ribosomal RNAs [17]. In cell lines, snoRNAs may act as either tumor suppressors or oncogenes, and the mechanisms underlying the various roles of snoRNA are an active area of investigation [18].

Peripheral blood cells in whole blood express up to 20,000 gene transcripts, which respond and adapt to micro- and macro-environmental pressures, thus representing a large pool of potential biomarkers for evaluation [19]. Peripheral blood gene expression profiles have been used in many tumor types, including RCC, to distinguish patients with malignancy from healthy subjects [20,21,22,23]. In the advanced and metastatic setting, peripheral blood mRNA analyses have identified prognostic genetic signatures in prostate [24,25], kidney [26], ovarian [27] and colon cancer [28].

Only one previous study by Burcynski et al. evaluated peripheral blood gene expression in advanced RCC and also observed elevation of ribosomal transcripts (ribosomal protein L4 and ribosomal protein L6) were associated with lower risk of death in advanced RCC [26]. When compared to the Burcynski study, in which 90% of blood samples were collected after at least one line of prior systemic therapy, all of our validation samples evaluated were obtained before any treatment was initiated, effectively eliminating changes in gene expression due to treatment effects. Additionally, while 25/45 patients included in the Burcinzksy study were positively identified as clear cell, all 47 in our validation cohort were clear cell RCC, minimizing potential differences in gene expression profiling in peripheral blood mononuclear cells due to inherent differences in tumor biology.

As with any study evaluating peripheral blood mRNA, an ongoing concern is controlling for external factors that might affect gene expression [29]. By ensuring a rapid and standard collection for isolating and storing RNA [30], we hope to maximize biologically meaningful changes in mRNA expression. One limitation common to gene expression studies is the lack of standardized housekeeping controls identified in whole blood [9]. However, gene expression studies in tissue samples in renal cell carcinoma and peripheral blood samples identified stably expressed genes that are suitable as housekeeping genes [8,10]. Finally, in the original article leading to the development to MSKCC score, the number of poor risk patients were 22%. Here, the number of patients in the poor risk MSKCC category were 4 (21%) in the DS and also 4 (8.5%) in the VS. With a relatively lower proportion of poor risk patients in the VS, further validation of these biomarkers in larger subsets is needed. Future steps include serial sampling of whole blood to identify novel predictive biomarkers, particularly for immune response.

## 4. Materials and Methods

Patient eligibility criteria: Between October 2011 and May 2015, patients with metastatic RCC were consented and samples were collected as part of a Mayo Institutional Review Board-approved biospecimen repository Protocol, MC 11-0855; “A Databank Study of Molecular Circulatory Biomarkers in Non-localized Renal and Testicular Cancer Patients” Date of initial approval-26 November 2011. All participants provided informed written consent prior to study participation. Samples were allowed to be collected from patients prior or during systemic treatment. Baseline patient characteristics, clinical stage, histopathologic features, MSKCC prognostic score, systemic treatments (when available) and survival data were included in the biorepository database.

RNA extraction and RNAseq library preparation: Blood specimens were collected in PAXgene RNA tubes (Qiagen NV, Venlo, The Netherlands) as has been previously published [31]. All samples used had RNA integrity numbers >8. RNA isolation and purification was performed using the PAXgene Blood RNA Kit per the manufacturer instructions (Qiagen NV). Whole-transcriptome RNA sequencing was performed on a discovery set (DS) of 19 randomly selected patients. RNA libraries were prepared using the TruSeq RNA Sample Prep Kit v2 (Illumina, San Diego, CA, USA). Libraries were sequenced as 51 × 2 paired-end reads on an Illumina HiSeq 2500 with TruSeq SBS v3 sequencing kits. After quality control analysis, RNA-seq data were processed by the Mayo Clinic Bioinformatics Core Facility (Supplementary Figure S1).

Bioinformatics and statistical analysis of RNAseq data: Reads were aligned using the GRCH38 reference genome with TopHat2 package utilizing Bowtie v2.2.3.0 as the underlying read-alignment software. Cuffdiff v2.2.1 was used to generate differential gene expression and analyzed via the cummeRbund package v2.10.0. Transcripts were measured using fragments per kilobase of transcript per million fragments mapped (FPKM). We calculated FPKM of each transcript and gene to identify correlations between the differentially expressed genes and MSKCC score. From this DS, candidate genes were selected for further prognostic performance determination based on magnitude fold change of enrichment (Supplementary Figure S2) and statistical significance in Cuffdiff, which reports statistical significance based on whether the *p*-value is greater than the false discovery rate (FDR) after applying the Benjamini–Hochberg correction 19. Additional details are provided in the Data Supplement.

Targeted RNA sequencing using NanoString: The selected candidate genes from the above “discovery set” were then explored in an independent cohort of patients with mCCRCC. We used the nCounter Analysis System (NanoString Technologies, Seattle, WA, USA) to quantify gene transcript expression from 18 target genes identified from the DS and 4 housekeeping genes (*ACTB*, *GADPH*, *HPRT1*, *TBP*) on a custom panel [32]. Selected housekeeping genes have previously been reported in the literature [8,9,10] and provide an average count as defined in nCounter Digital Analyzer as intermediate, medium and low coverage for the genes of interest. RNA extraction quality was assessed for use with NanoString, which recommends a 260/280 ratio of 1.9 or greater and a 260/230 ratio of 1.8 or greater. The nCounter Digital Analyzer was used to count individual fluorescent barcodes and quantify target RNA molecules present in each sample (Supplementary Mathods and Supplement Table S2).

Statistical analysis of NanoString data: The raw counts from the nCounter Analysis system were normalized to the geometric mean of 4 housekeeping genes. The software Cutoff Finder [11] was applied to identify optimal cutoffs for predicting overall survival (OS) defined as the time from initial metastatic stage to death or last follow-up. Cox proportional hazard regression and Kaplan–Meier analysis were performed on each target gene and the MSKCC for associations with OS using JMP Pro 10.0.0 (Cary, NC, USA). The Bonferroni correction for multiple biomarkers was applied, and only *p*-values < 0.0028 (α = 0.05/18 biomarkers) were considered statistically significant in univariate analysis. Time-dependent receiver operating characteristic (ROC) curve analysis was used to compare the predictive performance of each mRNA using Cutoff Finder [11].

## 5. Conclusions

Whole blood expression profiling in advanced clear cell RCC identifies several genes with clinically and statistically significant prognostic value. Evaluation of whole blood offers a non-invasive method of evaluating genomic biomarkers which enhance prognostic ability of conventional clinicopathologic models, including the MSKCC prognostic score.

## Figures and Tables

**Table 1:** Clinical and pathologic patient characteristics.

Variable	Category	Discovery Set (DS)	Validation Set (VS)
		RNA seq	NanoString
		*n* = 19	*n* = 47
Age (years)		71.6	66.4
(Median, Interquartile range (IQR)		(63.4, 79.3)	(62.5, 75.4)
Gender	Male	6	13
	Female	13	34
Histology	Clear cell	16	47
	Chromophobe	2	
	Papillary	1	
Fuhrman Grade (G)	G1		1
	G2	6	17
	G3	9	14
	G4	3	9
	Unspecified	1	6
Sarcomatoid differentiation	Present	0	5
	Absent	19	42
Clinical Stage at Initial Diagnosis	I	3	9
	II	4	9
	III	8	10
	IV	3	19
	Unspecified	1	
MSKCC prognostic score	1	7	31
	2	8	12
	3	4	4
Duration of follow-up among survivors in years (median, range)		4.28	3.6
		(1.34–4.47)	(0.06–4.12)
Number of deaths		11	25
Total lines of systemic therapy	0 (active monitoring)	3	7
	1	4	14
	2	4	5
	3	3	4
	4	3	1
	5 or more	2	3
	Unknown		13

**Table 2:** The 18 genes in the VS were analyzed using Cutoff Finder to find the optimal cutoff for predicting OS. Genes that reach statistical significance (*p* < 0.05) are emboldened.

		Univariate Analysis			Multivariate Analysis		
Gene	Cutoff	Hazard Ratio (HR)	95% CI	*p*-Value	HR	95% CI	*p*-Value
*AHSP*	977.9	0.51	0.23–1.13	0.091			
***BAG1***	**223.1**	**0.22**	**0.09–0.55**	**<0.0001**	**0.14**	**0.04–0.36**	**<0.0001**
*BLVRB*	4577	1.97	0.67–5.75	0.21			
*CA1*	3438	0.63	0.28–1.41	0.25			
*GMPR*	4093	0.58	0.26–1.32	0.19			
*GPX4*	1923	2.57	1.1–6.02	0.024			
*GUK1*	8740	1.72	0.64–4.64	0.27			
*HBM*	8640	0.48	0.16–1.42	0.18			
*HIPK3*	2652	1.95	0.8–4.8	0.14			
*HLAB*	36350	2.32	1.05–5.11	0.032			
*HLAC*	9550	2.88	0.86–9.67	0.074			
*LDHA*	1626	1.76	0.8–3.88	0.16			
***NOP56***	**545.3**	**0.23**	**0.1–0.53**	**<0.0001**	**0.13**	**0.05–0.34**	**<0.0001**
*PCGF5*	4361	0.42	0.14–1.24	0.11			
*PPDPF*	2303	0.51	0.17–1.5	0.21			
*PRDX5*	2315	3.55	1.26–10.0	0.012			
*SLC38A5*	2109	0.35	0.1–1.16	0.073			
*TCEB2*	132	0.38	0.11–1.29	0.11			

## Supplementary Materials

Supplementary materials can be found at www.mdpi.com/1422-0067/18/11/2326/s1.

## Abbreviations

AUC	Area under the curve
CI	Confidence Interval
FDR	False discovery rate
FPKM	Fragments per kilobase of transcripts per million fragments mapped
HR	Hazard ratio
mCCRCC	Metastatic clear cell renal carcinoma
mRCC	Metastatic renal cell carcinoma
mRNA	Messenger ribonucleic acid
MSKCC	Memorial Sloan Kettering Cancer Center
OS	Overall survival
RCC	Renal cell carcinoma
RNA	Ribonucleic acid
ROC	Receiver operating characteristic curve
