# Supplementary material for: Whole Blood mRNA Expression-Based Prognosis of Metastatic Renal Cell Carcinoma
Source: Int J Mol Sci. 2017 Nov 3;18(11):2326. doi: 10.3390/ijms18112326 (PMC5713295; doi:10.3390/ijms18112326)
Supplement: Supplementary file 1 [file ijms-18-02326-s001.pdf]

Supplementary methods

RNA-Seq alignment, differential gene expression and candidate gene selection

A total of 19 kidney samples were spliced aligned using TopHat v2.1.0 (TopHat2) [1] with three conditions defined as good, intermediate and poor with 7, 8, and 4 biological replicates, respectively. The number of pair-end reads mapped (R1 and R2) for all the replicates corresponded to 34.6 to 61.0 million above 97%. Supplementary table 1 summarizes alignment statistics. In this study we define biological replicates as parallel measurements of biological distinct samples of the same condition [2]. TopHat2 utilized Bowtie v2.2.3.0 as the underlying read-alignment software package. In this work all the alignments were performed against the Dec. 2013 (GRCh38/hg38) assembly of the human genome (hg38, GRCh38 Genome Reference Consortium Human Reference 38 (GCA_000001405.2). The annotation file corresponds to the GENCODE Release 23 (GRCh38.p3) [3]. The following options were used to run TopHat2: `'time tophat  -o tophat_output -p 4 -G $ANNOT $GENOME/hg38 $FASTQ'`, where time is the Linux command to print the total time that took TopHat2 to complete, $ANNOT corresponds to the transcript annotation file (GENCODE gene set) GRCh38GENCODEv23.gtf in General Transfer Format (gtf), $GENOME/hg38 corresponds to genome Bowtie2 index files generated using hg38.fa and finally $FASTQ is utilized to point to the location of the reads for each replicate. The results are stored in the accepted_hits.bam files that were used to run the next modules.

The next module that we employed for downstream analysis was cufflinks v2.2.1 to assemble transcripts, estimate their abundances, and test for differential expression and regulation in the above mentioned RNA-Seq samples [4-7]. These programs rely on the accepted_hitms.bam generated after running TopHat2. The options utilized with this module correspond to: `'time cufflinks -L selected_label  -p 4 $TOPHATDIR/accepted_hits.bam'` where –L is an option in cufflinks to allow labeling transcript fragments with a prefix "selected_label". We ran with 4 threads (-p 4) and used the output from TopHat2 as the input. We also used the script provided by cufflinks, namely, cuffmerge to combine novel isoforms and known isoforms and maximize overall assembly quality as stated in the manual [7].

The final step was to run cuffdiff v2.2.1 to generate differential gene expression [8]. Cuffdiff calculates gene expression for all the samples and provides information about statistical significance for the changes reported between samples [4]. The options selected were: `'time cuffdiff -o cuffdiff_out -b $GENOME/hg38.fa -p 8 -L G1,I1,P1 -u $MERGE/merged_asm/merged.gtf $SAMPLES'` where merged.gtf corresponds to the output from cuffmerge and $SAMPLES are all the accepted_hits.bam for conditions and replicates. Cuffmerge produces a gtf merged file from cufflinks transcript assemblies [8]. Cuffmerge merges transcript fragments from each sample into a comprehensive assembly [4]. The labels correspond to the three conditions Good (G1), Intermediate (I1), and Poor (P1), these labels are used throughout this paper. The Biomarker Discovery RNA-seq (BMD_RNA-seq) pipelineworkflow on the utilization of the above modules is illustrated in Figure S1. This workflow does not utilize any scripting language for communication between modules. It simplifies the swapping/elimination/addition of modules and follows the work reported in the literature [4].

All the data generated from this workflow was analyzed in the R environment via the cummeRbund package v2.10.0 [9] to render cuffdiff output in a graphical display. The following conventions were followed: all significant genes were obtained using the getSig() function with an alpha value of 0.05 [9]. Transcripts abundances were measured using fragments per kilobase of transcript per million fragments mapped (FPKM). A fragment corresponds to a single cDNA molecule and represented by a pair of reads at each end [7]. In addition, the base 2 log of the fold change between sample y and sample x, the uncorrected p-value and the false discovery rate (FDR) FDR-adjusted p-value were computed [4-8]. Cuffdiff reports the statistical significance based on whether p is greater than the FDR after applying the Benjamini-Hochberg correction [4-8]. Genes were selected by comparing the level of gene expression and the statistical significance [10]. Figure S2 shows a volcano plot to illustrate a pairwise comparison between the three conditions for all samples including all the replicates and all

genes. The red dots illustrate the set of genes that were considered significant when comparing fold change versus significance (-log p-values).

To be able to select gene markers that can discriminate between conditions the approached utilized by Cembrowski et al was selected [10]. A gene was considered X-fold enriched in a given condition, relative to other condition, when the FPKM value as reported by Cuffdiff was at least X-fold greater for all corresponding pairwise comparisons (e.g., for gene A to be X-fold enriched in G1 condition relative to I1 condition and P1 condition, $FPKM_{A,G1} > X \cdot FPKM_{A,I1}$ and $FPKM_{A,G1} > X \cdot FPKM_{A,P1}$. The set of genes with the largest enrichment fold and complying with statistical significance as defined by Cuffdiff were selected to be profiled as good candidates for gene markers between the three conditions previously defined. These top genes were compared for the three conditions and 18 genes were selected as gene markers to differentiate between conditions.

Validation of RNAseq data (NanoString)

The nCounter Digital Analyzer was used to count individual fluorescent barcodes to quantify gene expression. This technology is based on two probes. Capture probe linked to biotin molecule and reporter probe linked to a color-coded molecular marker. These probes hybridize to a complementary target mRNA using specific sequences from the genes of interest. These sequences are normally 100 bp in length. See Table S2 for gene positions and target sequences utilized in this study. The level of expression for the targeted genes was measured by image counting based on four different colors. The count correspond to the number of times a particular gene was detected [11]. We utilized 100 ng of total RNA isolated from fresh-frozen samples. The detailed protocol for mRNA quantification analysis is followed the manufacturer's recommendations, and are available at http://www.nanostring.com/uploads/Manual_Gene_Expression_Assay.pdf/ under http://www.nanostring.com/applications/subpage.asp?id=343. In addition, all the data generated with this technology was analyzed using the nCounter Digital Analyzer software, available at http://www.nanostring.com/support/ncounter/ [12].

References:

1. Kim, D.; Pertea, G.; Trapnell, C.; Pimentel, H.; Kelley, R.; Salzberg, S.L. Tophat2: Accurate alignment of transcriptomes in the presence of insertions, deletions and gene fusions. *Genome biology* **2013**, *14*, R36.
2. Blainey, P.; Krzywinski, M.; Altman, N. Points of significance: Replication. *Nature methods* **2014**, *11*, 879-880.
3. Harrow, J.; Denoeud, F.; Frankish, A.; Reymond, A.; Chen, C.-K.; Chrast, J.; Lagarde, J.; Gilbert, J.G.; Storey, R.; Swarbreck, D. Gencode: Producing a reference annotation for encode. *Genome biology* **2006**, *7*, S4.
4. Trapnell, C.; Roberts, A.; Goff, L.; Pertea, G.; Kim, D.; Kelley, D.R.; Pimentel, H.; Salzberg, S.L.; Rinn, J.L.; Pachter, L. Differential gene and transcript expression analysis of rna-seq experiments with tophat and cufflinks. *Nature protocols* **2012**, *7*, 562-578.
5. Roberts, A.; Pimentel, H.; Trapnell, C.; Pachter, L. Identification of novel transcripts in annotated genomes using rna-seq. *Bioinformatics* **2011**, *27*, 2325-2329.
6. Roberts, A.; Trapnell, C.; Donaghey, J.; Rinn, J.L.; Pachter, L. Improving rna-seq expression estimates by correcting for fragment bias. *Genome biology* **2011**, *12*, R22.
7. Trapnell, C.; Williams, B.A.; Pertea, G.; Mortazavi, A.; Kwan, G.; Van Baren, M.J.; Salzberg, S.L.; Wold, B.J.; Pachter, L. Transcript assembly and quantification by rna-seq reveals unannotated transcripts and isoform switching during cell differentiation. *Nature biotechnology* **2010**, *28*, 511-515.
8. Trapnell, C.; Hendrickson, D.G.; Sauvageau, M.; Goff, L.; Rinn, J.L.; Pachter, L. Differential analysis of gene regulation at transcript resolution with rna-seq. *Nature biotechnology* **2013**, *31*, 46-53.
9. Goff, L.; Trapnell, C.; Kelley, D. Cummerbund: Analysis, exploration, manipulation, and visualization of cufflinks high-throughput sequencing data. *R package version* **2013**, *2*.
10. Cembrowski, M.S.; Wang, L.; Sugino, K.; Shields, B.C.; Spruston, N. Hipposeq: A comprehensive rna-seq database of gene expression in hippocampal principal neurons. *eLife* **2016**, *5*, e14997.
11. Geiss, G.K.; Bumgarner, R.E.; Birditt, B.; Dahl, T.; Dowidar, N.; Dunaway, D.L.; Fell, H.P.; Ferree, S.; George, R.D.; Grogan, T. Direct multiplexed measurement of gene expression with color-coded probe pairs. *Nature biotechnology* **2008**, *26*, 317-325.

12. Reis, P.P.; Waldron, L.; Goswami, R.S.; Xu, W.; Xuan, Y.; Perez-Ordonez, B.; Gullane, P.; Irish, J.; Jurisica, I.; Kamel-Reid, S. Mrna transcript quantification in archival samples using multiplexed, color-coded probes. *BMC biotechnology* **2011**, *11*, 46.

**ID**	**RNA integrity number**	**R1 Mapped**	**R2 Mapped**	**Aligned with multiple alignments**	**Discordant alignments**
1	8.7	43447778 (98.3%)	43090775 (97.5%)	12530353 (29.4%)	1303242 (3.1%)
2	9.1	48485782 (98.6%)	48166072 (97.9%)	16903366 (35.5%)	915289 (1.9%)
3	8.6	34837128 (98.5%)	34634883 (97.9%)	11201466 (32.7%)	410811 (1.2%)
4	9.1	43759591 (98.6%)	43438843 (97.9%)	14231990 (33.1%)	605824 (1.4%)
5	8.9	45299621 (98.5%)	44862175 (97.5%)	10070050 (22.7%)	1246257 (2.8%)
6	9.0	42740410 (98.4%)	42369286 (97.5%)	10151142 (24.2%)	956422 (2.3%)
7	9.2	48077403 (98.6%)	47642274 (97.7%)	13564106 (28.8%)	1016240 (2.2%)
8	9.8	46097638 (98.4%)	45722975 (97.6%)	7802666 (18.1%)	1500722 (3.5%)
9	8.7	38124391 (98.0%)	37904817 (97.4%)	12481868 (33.4%)	814490 (2.2%)
10	9.2	40784150 (98.2%)	40566312 (97.7%)	12935320 (32.3%)	794282 (2.0%)
11	9.3	44250845 (97.6%)	44033476 (97.1%)	7802666 (18.1%)	1500722 (3.5%)
12	9.3	40518673 (97.9%)	40344894 (97.4%)	8242428 (20.8%)	1252513 (3.2%)
13	9.3	42463597 (98.2%)	41999160 (97.1%)	7844045 (18.9%)	1381792 (3.3%)
14	9.0	61038731 (98.5%)	60479805 (97.6%)	12690387 (21.2%)	1450512 (2.4%)
15	9.1	46499532 (98.7%)	46017786 (97.6%)	15640582 (34.3%)	574552 (1.3%)
16	9.3	43848630 (98.2%)	43401228 (97.2%)	14145401 (33.0%)	1193074 (2.8%)
17	9.0	44050504 (98.5%)	43588919 (97.4%)	16906169 (39.2%)	590628 (1.4%)
18	9.7	48395219 (98.2%)	47916093 (97.2%)	11756419 (24.9%)	1657063 (3.5%)
19	9.0	45571723 (98.3%)	45139835 (97.4%)	12500163 (28.0%)	1176822 (2.6%)

Supplemental Table S1. Alignment of pair-end reads mapped (R1 and R2) for all the replicates in the discovery set.

Gene	Accession	Position	Target Sequence
**ACTB***	NM_001101.2	1011-1110	TGCAGAAGGAGATCACTGCCCTGGCACCCAGCACAATGAAGATCAAGATCATTGCT CCTCCTGAGCGCAAGTACTCCGTGTGGATCGGCGGCTCCATCCT
**AHSP**	NM_016633.2	166-265	CAATGATCCTCTCGTCTCTGAAGAAGACATGGTGACTGTGGTGGAGGACTGGATGA ACTTCTACATCAACTATTACAGGCAGCAGGTGACAGGGGAGCCC
**BAG1**	NM_004323.3	1491-1590	CTCTTGTGATCGTGTAGTCCCATAGCTGTAAAACCAGAATCACCAGGAGGTTGCACC TAGTCAGGAATATTGGGAATGGCCTAGAACAAGGTGTTTGGCA
**BLVRB**	NM_000713.2	350-449	CTGCTGGGCACCCGCAATGACCTCAGTCCCACGACAGTGATGTCCGAGGGCGCCCG GAACATTGTGGCAGCCATGAAGGCTCATGGTGTGGACAAGGTCG
**CA1**	NM_001738.2	896-995	AAATGTTGAAGGTGATAACGCTGTCCCCATGCAGCACAACAACCGCCCAACCCAACC TCTGAAGGGCAGAACAGTGAGAGCTTCATTTTGATGATTCTGA
**GAPDH***	NM_002046.3	973-1072	CACTCCTCCACCTTTGACGCTGGGGCTGGCATTGCCCTCAACGACCACTTTGTCAAG CTCATTTCCTGGTATGACAACGAATTTGGCTACAGCAACAGGG
**GMPR**	NM_006877.3	326-425	CCATGTTTACAGCAATTCATAAGCATTACTCCCTGGATGACTGGAAGCTCTTTGCCAC AAATCACCCAGAATGCCTGCAGAATGTAGCCGTGAGTTCAGG
**GPX4**	NM_001039847.1	436-535	CAGGGAGTAACGAAGAGATCAAAGAGTTCGCCGCGGGCTACAACGTCAAATTCGAT ATGTTCAGCAAGATCTGCGTGAACGGGGACGACGCCCACCCGCT
**GUK1**	NM_000858.5	431-530	CGAGGCCCGGCGAGGAGAACGGCAAAGATTACTACTTTGTAACCAGGGAGGTGAT GCAGCGTGACATAGCAGCCGGCGACTTCATCGAGCATGCCGAGTT
**HBM**	NM_001003938.3	367-466	GACGAGTTCACCGTGCAAATGCAAGCGGCGTGGGACAAGTTCCTGACTGGTGTGGC CGTGGTGCTGACCGAAAAATACCGCTGAGCCCTGTGCTGCGCAG
**HIPK3**	NM_005734.2	2826-2925	TGAAGAGCAAGAAAGTAGTTGTGATACGGTGGATGGCTCTCCGACATCTGACTCTT CCGGGCATGACAGTCCATTTGCAGAGAGCACTTTTGTGGAGGAC
**HLA-B**	NM_005514.6	938-1037	CCCTGAGATGGGAGCCGTCTTCCCAGTCCACCGTCCCCATCGTGGGCATTGTTGCTG GCCTGGCTGTCCTAGCAGTTGTGGTCATCGGAGCTGTGGTCGC
**HLA-C**	NM_002117.4	896-995	AGCTGGGAGCCATCTTCCCAGCCCACCATCCCCATCATGGGCATCGTTGCTGGCCTG GCTGTCCTGGTTGTCCTAGCTGTCCTTGGAGCTGTGGTCACCG
**HPRT1***	NM_000194.1	241-340	TGTGATGAAGGAGATGGGAGGCCATCACATTGTAGCCCTCTGTGTGCTCAAGGGGG GCTATAAATTCTTTGCTGACCTGCTGGATTACATCAAAGCACTG
**LDH**	NM_001165414.1	1691-1790	AACTTCCTGGCTCCTTCACTGAACATGCCTAGTCCAACATTTTTTCCCAGTGAGTCAC ATCCTGGGATCCAGTGTATAAATCCAATATCATGTCTTGTGC
**NOP56**	NM_006392.2	606-705	TTCTCTATGCGTGTCAGGGAGTGGTACGGGTATCACTTTCCGGAGCTGGTGAAGAT CATCAACGACAATGCCACATACTGCCGTCTTGCCCAGTTTATTG
**PCGF5**	NM_001256549.1	183-282	GGAAAGCGGAACCACCAAAAGGAGTGATGATCAACGATCTCATGATAAATCTGGAT GCTAGTTCTCATGCCTCAGGACATCCTACTGGGAACGACACACC
**PPDPF**	NM_024299.2	289-388	ACCCGGGTCATTGGTGGGCCAGCTTCTTTTTCGGGAAGTCCACCCTCCCGTTCATGG CCACGGTGTTGGAGTCCGCAGAGCACTCGGAACCTCCCCAGGC
**PRDX5**	NM_012094.4	601-700	GGAAGGAGACAGACTTATTACTAGATGATTCGCTGGTGTCCATCTTTGGGAATCGA CGTCTCAAGAGGTTCTCCATGGTGGTACAGGATGGCATAGTGAA
**SLC38A5**	NM_033518.2	1300-1399	ACGACATGTGGCCATAGCTCTGATCCTGCTTGTTTTGGTCAATGTCCTTGTCATCTGT GTGCCAACCATCCGGGATATCTTTGGAGTTATCGGGTCCACC
**TBP***	NM_001172085.1	588-687	ACAGTGAATCTTGGTTGTAAACTTGACCTAAAGACCATTGCACTTCGTGCCCGAAAC GCCGAATATAATCCCAAGCGGTTTGCTGCGGTAATCATGAGGA
**TCEB2**	NM_007108.2	801-900	CTGCATGTCCACTCCCAGACGATGGCCAAGAGCAGAAACACAAGCTGGAGCCAGTG TCCTGGTTTGACAGCATGTTCAACGAGGGAACCCCAAGACGGAC

Supplemental Table S2: Genes, accession numbers, positions and targeted sequences used in NanoString codeset. *indicates housekeeping gene.

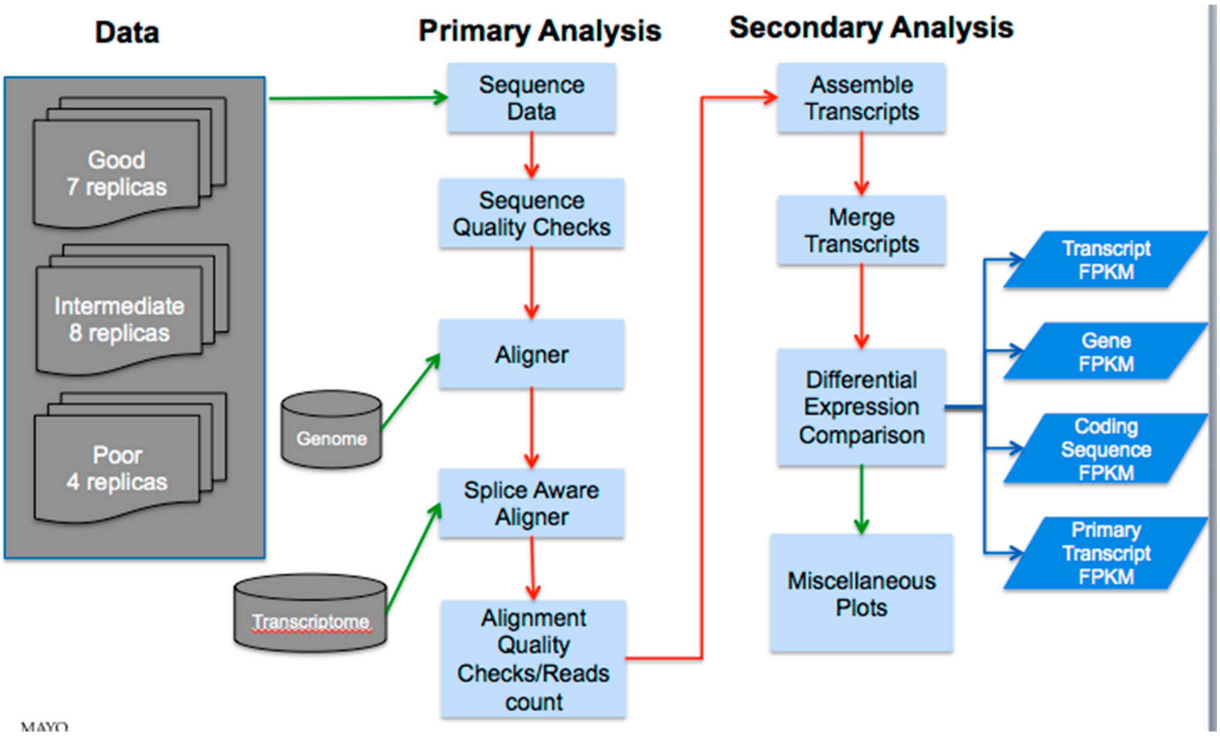

Supplemental Figure S1. Biomarker Discovery RNA-Seq pipeline utilized to perform primary and secondary analysis for 19 kidney samples.

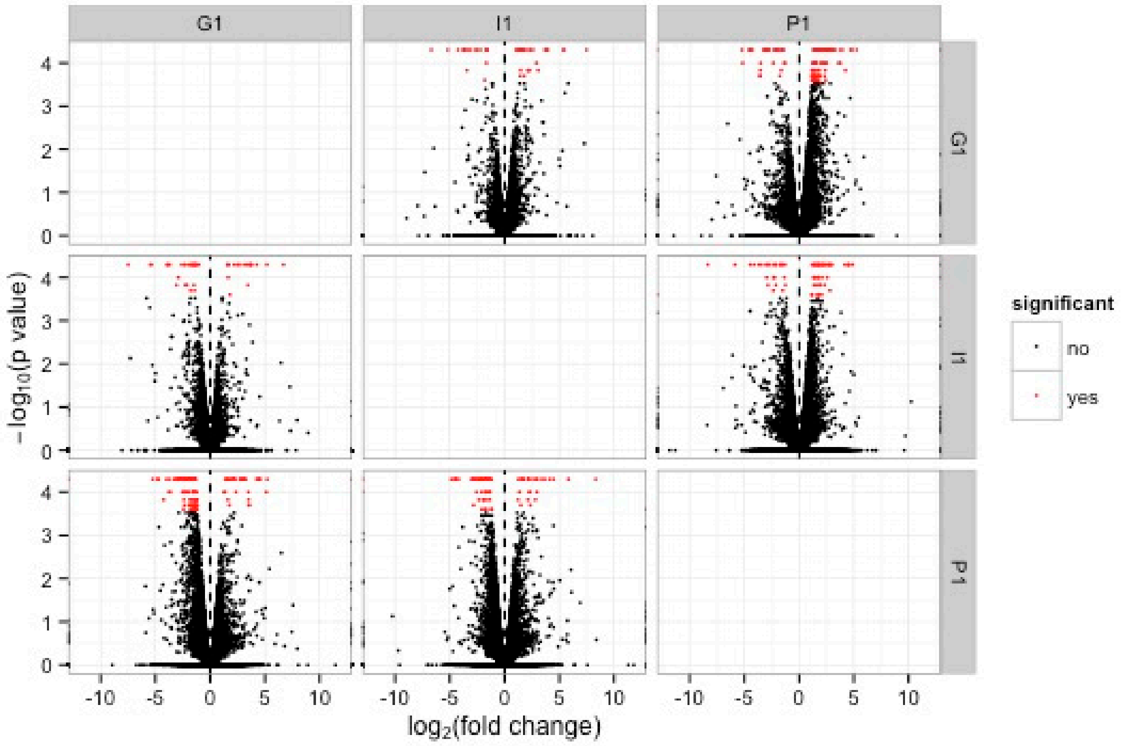

Supplemental Figure S2. Pairwise comparison between the three conditions (G1,I1,P1) for all samples including all the replicates and all genes. The red dots illustrate the set of genes that were considered significant when comparing fold change versus significance (-log p-values).
